# Pneumothorax and mortality in the mechanically ventilated SARS patients: a prospective clinical study

**DOI:** 10.1186/cc3736

**Published:** 2005-06-22

**Authors:** Hsin-Kuo Kao, Jia-Horng Wang, Chun-Sung Sung, Ying-Che Huang, Te-Cheng Lien

**Affiliations:** 1 Attending physician, Department of Respiratory Therapy, Taipei Veterans General Hospital; Department of Medicine, Taoyuan Veterans Hospital; National Yang-Ming University School of Medicine, Taipei, Taiwan; 2 Attending physician and Chief of Department, Department of Respiratory Therapy, Taipei Veterans General Hospital; National Yang-Ming University School of Medicine, Taipei, Taiwan; 3 Attending physician, Department of Anesthesiology, Taipei Veterans General Hospital; National Yang-Ming University School of Medicine, Taipei, Taiwan; 4 Attending physician, Department of Respiratory Therapy, Taipei Veterans General Hospital; National Yang-Ming University School of Medicine, Taipei, Taiwan

## Abstract

**Introduction:**

Pneumothorax often complicates the management of mechanically ventilated severe acute respiratory syndrome (SARS) patients in the isolation intensive care unit (ICU). We sought to determine whether pneumothoraces are induced by high ventilatory pressure or volume and if they are associated with mortality in mechanically ventilated SARS patients.

**Methods:**

We conducted a prospective, clinical study. Forty-one mechanically ventilated SARS patients were included in our study. All SARS patients were sedated and received mechanical ventilation in the isolation ICU.

**Results:**

The mechanically ventilated SARS patients were divided into two groups either with or without pneumothorax. Their demographic data, clinical characteristics, ventilatory variables such as positive end-expiratory pressure, peak inspiratory pressure, mean airway pressure, tidal volume, tidal volume per kilogram, respiratory rate and minute ventilation and the accumulated mortality rate at 30 days after mechanical ventilation were analyzed. There were no statistically significant differences in the pressures and volumes between the two groups, and the mortality was also similar between the groups. However, patients developing pneumothorax during mechanical ventilation frequently expressed higher respiratory rates on admission, and a lower PaO_2_/FiO_2 _ratio and higher PaCO_2 _level during hospitalization compared with those without pneumothorax.

**Conclusion:**

In our study, the SARS patients who suffered pneumothorax presented as more tachypnic on admission, and more pronounced hypoxemic and hypercapnic during hospitalization. These variables signaled a deterioration in respiratory function and could be indicators of developing pneumothorax during mechanical ventilation in the SARS patients. Meanwhile, meticulous respiratory therapy and monitoring were mandatory in these patients.

## Introduction

Severe acute respiratory syndrome (SARS) is a transmissible pulmonary infection caused by a novel coronavirus [1,2]. About 20 to 30% of SARS patients may progress to severe hypoxemic respiratory failure that requires mechanical ventilation and intensive care unit (ICU) admission [3-6]. Pneumothorax, a major and potentially lethal complication of SARS and mechanical ventilation, often complicates the management of mechanically ventilated patients, and would be especially hazardous for patients in an individually isolated SARS ICU. Peiris *et al*. identified a high incidence of pneumomediastinum (12%) in a general population of SARS patients [3]. In addition, Lew and Fowler also observed a high incidence of pneumothorax (20 to 34%) in mechanically ventilated SARS patients [6,7]. However, no further investigations have assessed the risk factors of pneumothorax in the mechanically ventilated SARS patients.

Patients with acute respiratory distress syndrome (ARDS) and acute lung injury (ALI) [8] developing pneumothorax have been extensively studied. Previous studies have found that high inspiratory airway pressure and positive end-expiratory pressure (PEEP) were correlated with barotraumas [9-11]. Eisner *et al*. analyzed a cohort of 718 patients with ALI/ARDS and revealed that higher PEEP was related to an increased risk of barotraumas [12]. However, others were unable to identify any relationship between barotrauma and high ventilatory pressure or volume in patients with early ARDS [13-15]. Therefore, the relationship between airway pressure or volume and the development of barotraumas remains uncertain.

To our knowledge, there is no study on the risk factors of pneumothorax in mechanically ventilated SARS patients. To address this issue, we performed a prospective study to determine whether pneumothorax was produced by high ventilatory pressure or volume, and if it was associated with an increased mortality rate at 30 days after mechanical ventilation.

## Materials and methods

This study included patients with SARS who were admitted to an isolation ICU at Taipei Veterans General Hospital. All patients satisfied the WHO case definition for SARS [16]. The research ethics board approved the study and we enrolled 41 patients with SARS who received mechanical ventilation between 14 May 2003 and 18 July 2003. Patients with pre-existing pneumothorax or chest tube thoracostomy were excluded. The primary study outcome variable was defined as radiographic evidence of new-onset pneumothorax at 30 days after ventilator use. Patients were censored at the first pneumothorax event, at the time of death, liberation from mechanical ventilation or discharge from the SARS ICU. Patients receiving mechanical ventilation were sedated with midazolam or propofol to facilitate mechanical ventilation; meanwhile, the sedatives were adjusted according to the Ramsay sedation score. Moreover, atracurium was used for neuromuscular paralysis to facilitate patient-ventilator synchrony in some patients. The dosage of atracurium was adjusted by peripheral nerve stimulator. When the patient was ready for weaning according to defined criteria, sedation and/or neuromuscular paralysis were discontinued.

Patient sex, age, actual body weight, APACHE II score and pre-existing comorbidities were recorded at entry. The PaO_2_/FiO_2 _ratio, PaO_2_, PaCO_2_, FiO_2 _and lung injury score [17] were recorded on ICU admission and daily during hospitalization. Ventilatory variables including PEEP, peak inspiratory pressure (PIP), mean airway pressure (MAP), tidal volume, tidal volume per kilogram, respiratory rate and minute ventilation were recorded at least once a day during the period of mechanical ventilation. When pneumothorax occurred, the highest pressure or volume of mechanical ventilation before the onset of pneumothorax were most likely to be the cause of pneumothorax [14]. Therefore, we compared the highest value of pressure and volume within a 24-hour period before the event in the patients with pneumothorax, with the overall values during mechanical ventilation in patients without pneumothorax.

Data were presented as mean ± standard deviation. The Mann-Whitney U test was used to compare data between patients with and without pneumothorax. We compared risk factors associated with the development of pneumothorax by Fisher's exact test for categorical variables. Non-parametric tests were chosen because of the small sample size in the pneumothorax group. Kaplan-Meier survival curves were compared by using the log-rank test. A *p *value of less than 0.05 was considered to indicate statistical significance. We used SPSS software (v10.0) for all analyses.

## Results

Demographic and clinical characteristics are shown in Table 1. Of the 41 patients, the male-to-female ratio was 1:0.37 and mean age was 75.4 years. Five patients developed pneumothorax and the incidence of pneumothorax was 12%. The mean time to the development of pneumothorax was 8.0 ± 4.4 days after ventilator use. Of the patients, 28 (68%) met the criteria for either ALI or ARDS. Patients with pneumothorax were significantly associated with higher respiratory rate on admission, and more pronounced hypoxemia with lower PaO_2_/FiO_2 _ratio and higher PaCO_2 _during hospitalization.

Table 2 compares ventilator variables according to the presence or absence of pneumothorax. There were no significant differences in any pressure or volume between the patients with and without pneumothorax.

**Figure 1:**
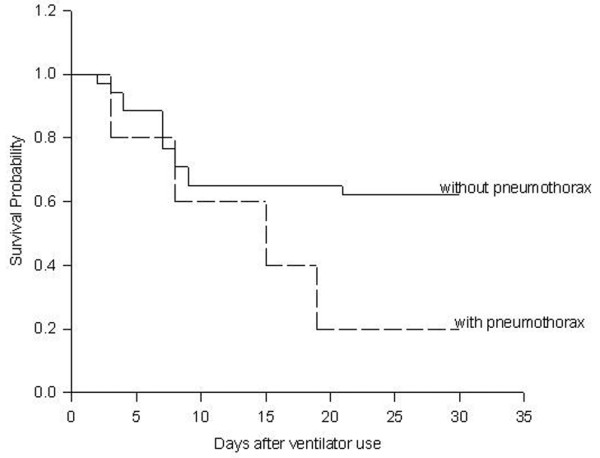
Kaplan-Meier curve of the probability of survival over time for mechanically ventilated SARS patients. (*p *= 0.11).

The overall survival rate was 59% at 30 days after mechanical ventilation. The relationship between pneumothorax and the probability of survival is shown in Fig. 1. There were no significant differences between the patients with and without pneumothorax.

## Discussion

In the present study, we focused on the mechanically ventilated SARS patients and analyzed the risk factors of pneumothorax. Our study demonstrated that mechanically ventilated SARS patients with higher baseline respiratory rate, lower PaO_2_/FiO_2 _ratio, and higher PaCO_2 _during hospitalization were at a greater risk of developing pneumothorax. There were no significant differences in pressure, volume and mortality rate between the patients without and with pneumothorax. Barotrauma is a common complication in patients with SARS. The previous study by Peiris identified a high incidence of pneumomediastinum (12%) in a general population of SARS patients [3]. Choi *et al*. had also shown that subcutaneous emphysema, pneumothorax and pneumomediastinum were detected in six SARS patients (2.2%) who had not received positive-pressure ventilation [18].

In our study, the incidence of pneumothorax in mechanically ventilated SARS patients was lower than previous studies (12% versus 20 to 34%) [6,7]. The incidence of barotrauma in patients with ALI/ARDS varies widely. In most recent studies, it has ranged from 5 to 15% [12,14,19]. Gammon and colleagues have shown that the presence of ARDS is the major independent risk factor of barotraumas [13,20]. This may explain the lower incidence of pneumothorax in our study since the proportion of our patients with ALI/ARDS (68%) is lower than the other studies [6,7].

Another important finding in our study was the lack of correlation between ventilator variables and the presence of pneumothorax. Our results agreed with most of the previous studies that were done on ARDS patients. In the ARDS Network randomized controlled trial, low tidal volume ventilation decreased mortality without influencing the incidence of barotraumas [19]. In patients with sepsis-induced ARDS, there were no significant correlations between the ventilatory parameters and the development of pneumothorax or another air leak [14]. These authors suggested that barotrauma was more related to the underlying process than to the ventilator settings [14,15].

We found that the mechanically ventilated SARS patients with pneumothorax had a significant baseline tachypnea. Additionally, patients with a higher respiratory rate on admission also showed a trend of higher respiratory rate during hospitalization. (*p *= 0.06). Tachypnea on admission probably reflected the increased severity of the underlying disease [21], which may directly lead to a higher incidence of pneumothorax. There was also a higher risk of auto-PEEP in patients with tachypnea due to insufficient expiratory time, which may also contribute to the development of pneumothorax. However, auto-PEEP was not recorded in this study.

In our study, SARS patients with pneumothorax had a higher PaCO_2 _during hospitalization. Gattinoni *et al*. also observed a similar finding in ARDS patients with pneumothorax [11]. Increased dead space and cystic changes of lung parenchyma due to worsening underlying disease played a major role in patients with hypercapnia. This mechanism is further supported by a thin-section computed tomographic study that was done by Joynt and colleagues on the late stage of ARDS (more than 2 weeks after onset) caused by SARS [22]. They found that severe SARS-induced ARDS might independently result in cyst formation. In our study, patients with pneumothorax were also associated with a more pronounced hypoxemia, with lower PaO_2_/FiO_2_ during hospitalization compared with those without pneumothorax (65.8 versus 210.1). Oxygen-diffusing impairment and ventilation-perfusion maldistribution may play a role in developing hypoxemia in the mechanically ventilated SARS patient. A decrease in PaO_2_/FiO_2 _and increase in PaCO_2 _may be considered as a deterioration of respiratory condition in a patient with ALI/ARDS. The presence of pneumothorax together with hypoxemia/hypercapnia may indicate worsening of the underlying disease. This is supported by the large difference in APACHE II (26.0 ± 11.8 versus 20.7 ± 6.6) and ALI (2.51 ± 0.29 versus 1.59 ± 1.10) scores between patients with and without pneumothorax in this study, although these did not reach statistical significance.

In our study, the mortality rate was not significantly increased in patients with pneumothorax. In other studies on ALI/ARDS, the mortality directly attributable to barotrauma was low [12,14,23]. The mortality rate was 41% in our study, which was higher than the 26% from the results of five cohort studies [2-4,24,25]. Older age and more comorbidities may be the major causes. Age and coexisting illness, especially diabetes mellitus and heart disease, were consistently found to be independent prognostic factors for the risk of death and the need for intensive care in SARS patients [3-5,26,27].

There are several limitations to our study. Data were recorded once daily in individual isolation rooms and may have missed transient elevations in airway pressure/volume that could have led to alveolar disruption and pneumothorax. Secondly, we selected parameters that were easily measured and were previously shown or theorized to contribute to alveolar disruption, including ventilator variables and high-risk disease states. However, it is possible that an important variable such as plateau pressure was omitted from this analysis. Thirdly, there were only 41 mechanically ventilated SARS patients in our study. A study with a larger sample size may demonstrate statistical significance. The above factors are likely to cloud the relationship between the ventilatory variables and the occurrence of barotrauma.

## Conclusion

The analysis of pneumothorax in mechanically ventilated SARS patients indicates that the patients with higher respiratory rates on admission, and lower PaO_2_/FiO_2 _ratio and higher PaCO_2 _during hospitalization had a greater risk of pneumothorax. The correlation between the clinical characteristics and pneumothorax may be considered as a deterioration of respiratory function in mechanically ventilated SARS patients developing pneumothorax. Pneumothorax in mechanically ventilated SARS patients may be an indicator of worsening underlying lung disease.

## Key messages

• 	There were no significant differences in pressure, volume and mortality rate between the mechanically ventilated SARS patients without or with pneumothorax.

• 	Mechanically ventilated SARS patients with higher baseline respiratory rate, lower PaO_2_/FiO_2 _ratio, and higher PaCO_2 _during hospitalization were at a greater risk of developing pneumothorax.

• 	The correlation between the clinical characteristics and pneumothorax may be considered as a deterioration of respiratory function in mechanically ventilated SARS patients developing pneumothorax.

## Abbreviations

ALI = acute lung injury; APACHE = Acute Physiology and Chronic Health Evaluation; ARDS = acute respiratory distress syndrome; FiO_2 _= fraction of inspired oxygen; MAP = mean airway pressure; ICU = intensive care unit; PEEP = positive end-expiratory pressure; PIP = peak inspiratory pressure,  SARS = severe acute respiratory syndrome.

## Competing interests

The author(s) declare that they have no competing interests.

## Authors' contributions

T-CL participated in the design of the study and performed the statistical analysis. H-KK made contributions to the collection, analysis and interpretation of data. J-HW, C-SS and Y-CH made contributions to the design of the study and performed the statistical analysis.

## Figures and Tables

**Table 1:** Demographic and clinical characteristics according to the presence or absence of pneumothorax

Variable	No pneumothorax	Pneumothorax	*p *value
Number of patients (%)	36 (88)	5 (12)	
Gender (male/female)	26/10	4/1	1
Age, years	76.3 ± 10.4	68.8 ± 18.0	0.46
Body weight, kg	58.5 ± 12.4	57.0 ± 18.2	0.98
APACHE II score	20.7 ± 6.6	26.0 ± 11.8	0.41
Pre-existing comorbidities			
Chronic renal insufficiency	4	0	1
Congestive heart failure	9	2	0.59
Diabetes mellitus	15	2	1
Chronic obstructive pulmonary disease	5	0	1
Pulmonary tuberculosis	2	2	0.06
Cerebrovascular disease	17	1	0.37
On ICU admission			
Baseline lung injury score	1.27 ± 1.04	1.59 ± 0.59	0.35
Baseline respiratory rate	25.32 ± 7.53	36.00 ± 5.10	0.006
Baseline PaO_2_/FiO_2 _ratio	289.9 ± 172.9	272.6 ± 140.8	0.87
Baseline PaCO_2_	35.7 ± 9.3	49.4 ± 23.0	0.20
During hospitalization			
Highest lung injury score	1.59 ± 1.10	2.51 ± 0.29	0.09
Highest respiratory rate	34.65 ± 5.19	40.80 ± 7.08	0.06
Lowest PaO_2_/FiO_2 _ratio	210.1 ± 123.8	65.8 ± 24.3	0.02
Highest PaCO_2_	49.9 ± 17.4	80.1 ± 12.3	0.004
ALI/ARDS (%)	24 (66%)	4 (80%)	1
Liberation from ventilator (%) at 30 days	11(31)	0	0.29

Data are presented as mean ± standard deviation. ALI, acute lung injury; APACHE, Acute Physiology and Chronic Health Evaluation; ARDS, acute respiratory distress syndrome; FiO_2_, fraction of inspired oxygen; ICU, intensive care unit; PEEP, positive end-expiratory pressure.

**Table 2:** The ventilator variables according to the presence or absence of pneumothorax

Variables	No pneumothorax	Pneumothorax	*p*
Ventilatory pressure, cmH_2_O, or volume			
positive end-expiratory pressure	7.94 ± 4.38	8.2 ± 2.0	0.54
peak inspiratory pressure	34.78 ± 6.80	33.8 ± 3.76	0.73
mean airway pressure	18.75 ± 4.89	20.8 ± 1.78	0.17
tidal volume, ml	761.02 ± 128.87	733.8 ± 154.0	0.43
tidal volume/kg, ml	12.32 ± 2.71	12.54 ± 3.34	0.97
Minute ventilation, l (on ICU admission)	10.40 ± 3.00	11.38 ± 2.84	0.34
Minute ventilation, l (during hospitalization)	15.33 ± 4.68	12.93 ± 4.10	0.26

Data are presented as mean ± standard deviation.
